# Biomarkers of An Autoimmune Skin Disease—Psoriasis

**DOI:** 10.1016/j.gpb.2015.04.002

**Published:** 2015-09-08

**Authors:** Shan Jiang, Taylor E. Hinchliffe, Tianfu Wu

**Affiliations:** 1 Department of Dermatology, Renmin Hospital of Wuhan University, Wuhan 430060, China; 2 Department of Biomedical Engineering, University of Houston, Houston, TX 77204, USA

**Keywords:** Psoriasis, Biomarker, Genomics, Proteomics, Metabolomics

## Abstract

**Psoriasis** is one of the most prevalent autoimmune skin diseases. However, its etiology and pathogenesis are still unclear. Over the last decade, omics-based technologies have been extensively utilized for **biomarker** discovery. As a result, some promising markers for **psoriasis** have been identified at the **genome**, transcriptome, **proteome**, and **metabolome** level. These discoveries have provided new insights into the underlying molecular mechanisms and signaling pathways in **psoriasis** pathogenesis. More importantly, some of these markers may prove useful in the diagnosis of **psoriasis** and in the prediction of disease progression once they have been validated. In this review, we summarize the most recent findings in **psoriasis biomarker** discovery. In addition, we will discuss several emerging technologies and their potential for novel **biomarker** discovery and diagnostics for **psoriasis**.

## Introduction

**Figure 1:**
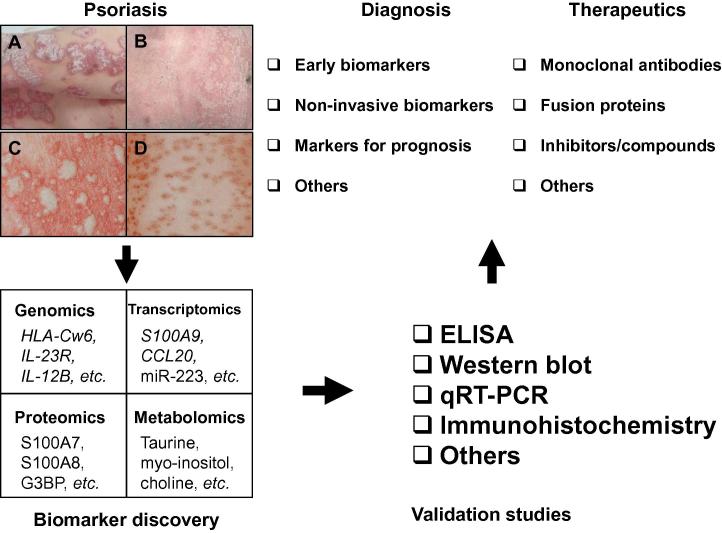
**Pipeline of biomarker discovery and therapeutic targeting in psoriasis** Psoriasis is one of the most prevalent autoimmune inflammatory skin diseases. The four main clinical types are plaque (vulgaris) (**A**), erythrodermic (**B**), pustular (**C**), and guttate (**D**). Potential biomarkers of psoriasis could be identified using various technologies including genomics, transcriptomics, proteomics, and metabolomics. Some promising biomarkers of psoriasis have been identified with different “omics” platforms as shown in the figure, and more exciting findings could be expected with the advancement of these technologies. Valuable biomarkers should be validated using orthogonal techniques such as ELISA, Western blot, qRT-PCR, and IHC with a larger cohort of subjects, in order to achieve statistically meaningful results. The validated biomarkers could potentially be useful in the clinical diagnostics and therapeutics of psoriasis.

Psoriasis is a common, chronic, and recurrent autoimmune inflammatory skin disease, affecting approximately 2% of the population in the United States [1]. Psoriasis generally manifests as chronic inflammation of the skin and is characterized by circumscribed, scaling, and erythematous plaques. Recurrent episodes occur during a patient’s lifetime, which often can be improved through treatment, with few spontaneous remissions. Psoriasis vulgaris (also called plaque psoriasis) is the most common form of the disease, affecting 85%–90% of the patients [2]. Other types of psoriasis include erythrodermic psoriasis, guttate psoriasis, and pustular psoriasis (Figure 1). Although psoriasis is considered a skin disease, patients could develop comorbidities, including psoriatic arthritis (PsA), metabolic syndromes, and cardiovascular diseases [3], in addition to skin lesions.

Previous studies on the pathogenic factors and immune mediators of psoriasis have greatly advanced our understanding of disease pathogenesis. Accumulating clinical and experimental evidence points out that the immune system plays a key and central role in disease pathogenesis. Psoriasis has been considered a T helper type 1 (Th1)-mediated disease for many years [4] and recent studies have demonstrated that the interleukin (IL)-23/Th17 cell axis plays a crucial role in psoriasis pathogenesis [5]. In the initiation phase, keratinocytes release antimicrobial peptide LL37 after trauma or infection, which can bind to self-DNA and self-RNA fragments that are released by dying or stressed skin cells [6]. These complexes activate plasmacytoid dendritic cells (pDCs) to produce type I interferons (IFN), like IFN-α [6]. In turn, type I IFNs and immune complexes can activate myeloid DCs (mDCs) through Toll-like receptor 8 (TLR8). IL-23 and IL-12 that are released from activated mDCs can then activate Th17, Th1, and Th22 cells to produce an abundance of cytokines, such as IL-17, IL-22, IFN-γ, and tumor necrosis factor (TNF). These cytokines help to stimulate the keratinocytes to amplify the inflammation typically observed in psoriatic lesions [7].

The diagnosis of psoriasis is primarily focused around the clinical morphologic evaluation of a skin lesion, as there are no other clearly-defined diagnostic criteria. The differential diagnosis of psoriasis is abundant and depends on the clinical subtype. Histopathological analysis of a skin biopsy specimen is currently the most common and efficient clinical identification method. Nonetheless, skin biopsy is invasive and the pathological alterations are not obvious at early stages of psoriasis. Therefore, there is an urgent need to develop non-invasive diagnostic tests or biomarkers with high sensitivity and specificity for psoriasis [8].

Although there is no cure for psoriasis, some biological therapies targeting specific immune components have recently proven to be highly effective [9]. Earlier biological agents, including efalizumab and alefacept, primarily disrupt the activation and migration of T cells, whereas agents like infliximab, etanercept, and adalimumab target TNF-α. Recently, agents like ustekinumab and ABT-874, which target the p40 subunit shared by both IL-12 and IL-23, have been developed, as well as new anti-IL-17 agents and anti-IL-23p19 agents [9,10]. However, approximately 20%–30% of psoriasis patients fail to respond to biological therapies [8]. Therefore, valuable biomarkers for the diagnosis, prognosis, and treatment of psoriasis are of great significance for clinicians in designing effective and personalized therapies.

In this review, we will summarize the most up-to-date research findings in biomarker discoveries for psoriasis, including biomarkers identified with conventional technologies, genomic biomarkers, transcriptomic biomarkers, proteomic biomarkers, and metabolomic biomarkers. In addition, we will discuss several emerging technologies, which have potentials in novel biomarker discovery validation and diagnostics in psoriasis.

## Biomarkers identified with conventional technologies

Conventional assays, such as bioplex assays, ELISA, Western blotting, and immunohistochemistry (IHC), have been used to identify potential biomarkers for psoriasis. Early studies have found that serum levels of nonspecific inflammation markers, including C-reactive protein (CRP), platelet P-selectin, haptoglobin, complement component 3 (C3), and C4 [11,12], as well as some pro-inflammatory cytokines, such as TNF-α, IFN-γ, IL-6, IL-8, IL-12, and IL-18, were increased in psoriasis patients [8]. However, no elevated serum IL-17A levels were detected in different cohorts of psoriatic patients, although Th17 cells, which produce IL-17, were noted to play an important role in psoriasis [8]. This may simply be due to low serum levels of IL-17A, low sensitivity of the assays or other unknown reasons. Psoriasis patients also exhibited abnormalities in blood fibrinolysis and coagulation, such as increased levels of fibrinopeptide A, fibrinogen, D-dimer, and C4, in addition to decreased levels of protein C, alpha 2-antiplasmin, and plasminogen [13].

Nowadays, psoriasis has been increasingly viewed as a systemic disease that is associated with metabolic syndrome and/or its constituent pathologies, which may include insulin resistance, obesity, atherogenic dyslipidemia, and hypertension [14]. There are also various abnormalities in lipid metabolism, as well as oxidative stress in psoriasis patients [15]. High levels of lipids, *e.g.*, total cholesterol, triglycerides, low-density lipoprotein (LDL), cholesterol, and very low-density lipoprotein (VLDL), in the blood and lipid peroxidation, as well as decreased anti-oxidant enzyme activity were found in psoriasis patients [15–17]. Specifically in psoriatic epidermis, levels of total lipids, oxidized LDL, phospholipids, triacylglycerols, and cholesterol were shown to be notably increased [18]. High levels of oxidized LDL in both the skin and blood may account for both psoriasis pathogenesis and the risk of developing atherosclerosis [18]. Overall, an imbalanced oxidative status influences cell proliferation, differentiation, and apoptosis in psoriasis [8].

Most biomarkers that are differentially expressed in psoriatic skin tissue are related to abnormal keratinocyte differentiation and proliferation [8]. The expression of hyperproliferation markers, such as keratin 6 (K6) and K16, was up-regulated, whereas expression of terminal differentiation markers, such as K1 and K10, was down-regulated in psoriatic epidermis [19]. In psoriatic skin, amounts of p53, antigen Ki67, heat shock proteins (HSP60), connexin 26 (Cx26), and Cx30 were up-regulated, which contribute to epidermal hyperproliferation [20]. In psoriatic skin, a significant reduction in protein expression of B-cell lymphoma 2 (Bcl-2) was found, whereas there was a considerable degree of overexpression of Bcl2-associated X protein (Bax) and Bcl-extra large (Bcl-xL), which is correlated with responses to anthralin and anti-TNF therapy [20–22]. In line with the inflammatory nature of the disease, an imbalanced cytokine milieu has been found in psoriatic lesions, with reduced levels of IL-1, IL-4, IL-5, and IL-10, together with increased levels of TNF-α, IFN-α, IL-2, IL-6, IL-8, IL-12, IL-23, IL-23R, and LIF-1 [8]. IL-33 is a novel member of the IL-1 superfamily of cytokines, and its expression is up-regulated following pro-inflammatory stimulation [23]. There was significantly higher *IL-33* gene expression and protein expression in psoriatic skin lesions than in normal control skin [23]. Recently, DaErme et al. [24] described a group of IL-17/TNFα-associated genes with expression profiles that were specific to psoriatic skin. Among them, IL-36γ proved to be the most notable marker. IL-36γ was expressed only in psoriasis lesions and the serum level of IL-36γ in the peripheral blood was closely associated with disease activity [24].

## Genomic biomarkers

Psoriasis is a complex genetic disease, which can be attributed to the interaction of multiple genetic and environmental factors [25]. At present, at least 13 major psoriasis susceptibility loci (*PSORS1-13*) have been described, originally based on family-based linkage disequilibrium (LD) studies [26]. The major histocompatibility complex (MHC) class I is identified as a major susceptibility factor in psoriasis [27]. The gene located at Chromosome 6p21 is primarily associated with the development of psoriasis, and has been documented as *PSORS1* [28]. *PSORS1* is the strongest susceptibility locus, which is thought to account for approximately 35%–50% of the heritability of psoriasis [26]. Within *PSORS1*, *HLA-Cw6* is the primary allele associated with psoriasis [26]. Clinical subgroups of psoriasis have different genetic heterogeneity at *PSORS1*. For instance, early onset and guttate psoriasis is strongly associated with *PSORS1*, whereas late onset (occurring cases in individuals aged more than 50 years) and palmoplantar psoriasis is not [29].

Recent genetic studies, such as analyzing single nucleotide polymorphisms (SNPs) as genetic markers, systematic mapping of human haplotypes, and developing high performance genotyping platforms, have created an enabling framework for genome-wide association studies (GWAS). Using GWAS, strong associations of disease phenotypes with the *PSORS1* region have been confirmed, and new associated genes other than MHC have also been identified (Table 1). Except the *IL12B*, *IL23R*, and *IL-23A* variants [30,31], these include genes encoding zinc-finger protein 313 (*ZNF313*) [32], TNFAIP3-interacting protein1 (*TNIP1*) [33], and TNF-α-induced protein 3 (*TNFAIP3*) [33] within the nuclear factor κB (NF-κB) pathway, as well as a genetic region that is believed to be involved in the regulation of the innate immune system and apoptosis.

Recently, as a result of a meta-analysis composed of three GWAS alongside two independent datasets that were genotyped on the immunochip, 15 new psoriasis susceptibility regions have been identified in Caucasians [34]. The newly-identified disease regions encompassed some genes encoding proteins that regulate T-cell function, such as *RUNX3*, *STAT3*, and *TAGAP* [34]. Other notable candidate genes included those involved in macrophage activation (*ZC3H12C*), NF-κB signaling (*CARD14* and *CARM1*), and IFN-mediated antiviral responses (*DDX58*) [34].

A GWAS examining both SNP and copy number variants (CNV) identified that the *late cornified envelope* (*LCE*) gene cluster was strongly associated with psoriasis [35]. β-Defensins may be another potential genetic marker for psoriasis [36]. β-Defensins are small, antimicrobial peptides that are secreted in the epidermis in order to guard against microbial invasion [36]. Significant associations between higher genomic copy number for the β-defensin gene cluster and the risk of psoriasis was found in a Dutch and German cohort [36].

These genomic biomarkers have provided some insights into the potential mechanisms that trigger the psoriasis phenotype in genetically-susceptible individuals. Most of these susceptibility genes are involved in immunological and inflammatory processes, further supporting a central role of the immune system in psoriasis pathogenesis.

## Transcriptomic biomarkers

The first psoriasis-associated transcriptome was reported in 2001 using an early Affymetrix platform (HuGeneFL), encompassing 159 genes [37]. Of these 159 genes, transcript levels were significantly altered in patients who responded to therapeutic intervention, and a good deal of the changes in gene expression arose prior to visible clinical improvement [37]. Following therapeutic intervention using an immunomodulatory cytokine, recombinant human IL-11 (rhIL-11), or an immunosuppressant, cyclosporine A, the expression of a subset of 41 genes, which was shown to be differentially regulated between lesional and uninvolved skin, was restored to normal levels [37]. Among them, 10 genes including the inhibitor of DNA binding 4 (*ID4*), heparin binding protein 17 (*HBP-17*), keratin 16 (*KRT16*), *S100A2*, *S100A9*, *S100A12*, guanine nucleotide binding protein 15 (*GNA15*), *MTX*, *PRKMK3*, and *SCYA2* were all found to be localized in the psoriasis susceptibility loci [37]. These studies provide a group of candidate genes that could serve as both targets for novel therapeutic intervention and surrogate/predictive markers for treatment outcome.

Gudjonsson et al. [38] identified 1326 differentially-regulated transcripts for 918 unique genes using RNA microarrays (Affymetrix, HU133 plus 2.0 arrays). The significantly-upregulated genes included *DEFB4*, *PI3*, and *SERPINB4*, as well as several S100 family members. On the other hand, significantly-downregulated genes included those encoding the Wnt-inhibitory factor-1 (WIF1), CCL27, and betacellulin (BTC) [38]. Enriched gene ontology (GO) categories included immune responses, keratinocyte differentiation, and defense responses.

Alterations in gene expression happen at the early stage of psoriasis, even before the psoriatic skin lesions occur [31]. By examining transcripts with notably-altered (>1.3-fold) expression via gene cluster analysis, a group of genes were identified. These include genes encoding peroxisome proliferator-activator receptor alpha (PPARα), sterol regulatory element-binding protein (SREBF), and estrogen receptor 2 (ESR2). These genes displayed a highly-correlated expression pattern and are involved in lipid metabolism. Dramatic alterations in expression of these transcription factors point toward a ‘pre-psoriatic’ signature, which is characterized by increased innate immunity and decreased lipid biosynthesis, compared to normal skin [31].

A recent study using RNA microarrays (Affymetrix, HU133 plus 2.0 arrays) by Suárez-Fariñas et al. [39] found 4175 differentially-expressed transcripts in psoriasis lesions versus non-lesion samples. Approximately 60% of the top 20 upregulated genes, such as *S100A12*, *SPRR2C*, and *CXCL1*, have additive or synergistic responses to IL-17 and TNF, suggesting that these cytokines will be important for the creation of a molecular profile for psoriasis. Many of the upregulated genes that are involved in signaling pathways, including the IFN-γ, IL-17, and TNF signaling pathways, might be central to the pathogenesis of psoriasis. For instance, some of the upregulated genes, such as *OASL*, *CXCL1*, *STAT-1*, and *Mx-1*, belong to the IFN-γ signaling pathway; whereas *CCL20* and *CXCL8* (*IL-8*) belong to the IL-17 signaling pathway; and *AKR1B10*, *IL1F9*, and *CXCL9* belong to the TNF signaling pathway [39]. In addition, *rennin*, a gene known to be involved in the renin–angiotensin signaling pathway, has been confirmed to be differentially expressed in psoriasis skin lesions [39]. Such genes link psoriasis to metabolic disease pathways as well as to the cardiovascular risk pathway. Moreover, by combining laser capture microdissection with microarray analysis of the epidermal and dermal skin compartment, Mitsui et al. [40] have identified locally-expressed psoriasis-relevant genes in psoriatic dermis, such as genes encoding CCL19 and its receptor CCR7.

Transcriptional changes in psoriasis occur during epidermal differentiation and keratinization. These analyses of differentially-regulated transcripts may provide additional insights into the molecular mechanisms and signaling pathways involved in psoriasis pathogenesis.

## Epigenetic biomarkers

Epigenetic mechanisms, such as DNA methylation, microRNA (miRNA) or long non-coding RNA (lncRNAs) expression, and histone modifications, could cause alterations in gene expression and chromatin remodeling. These represent plausible linkers between environmental exposure and psoriasis [41].

miRNAs are post-transcriptional regulators that bind to complementary sequences in the 3′ UTRs of mRNAs. miRNAs can lead to target gene silencing, and their levels in serum can be useful biomarkers for diagnosis and prognosis with additional therapeutic value in various diseases. Serum levels of miR-1266 were significantly higher in patients with psoriasis, as compared to healthy control subjects [42]. Furthermore, serum level of miR-1266 displayed a weak inverse correlation with the psoriasis area severity index (PASI) score, as well as body surface area of the involved skin [42]. Moreover, the global expression of miR-223 and miR-143 in peripheral blood mononuclear cells (PBMCs) from psoriasis patients was positively correlated with the PASI score [43]. Receiver-operating characteristic analysis (ROC) showed that both miR-223 and miR-143 may be capable of distinguishing psoriasis patients from healthy controls [43]. Interestingly, after treatment with methotrexate (MTX) for 3–5 weeks, expression of miR-223 and miR-143 was significantly downregulated in the PBMCs from psoriasis patients, following a significant decrease in psoriasis severity [43]. Some highly-upregulated miRNAs in psoriatic skin lesions include hematopoietic-specific miRNAs, such as miR-142-3p and miR-223/223, angiogenic miRNAs, such as miR-21, miR-378, miR-100, and miR-31, as well as epithelial differentiation miRNAs, such as miR-203 [44]. miR-203 targets the *SOCS-3* gene coding for suppressor of cytokine signaling 3, which is a negative regulator of the STAT-3 pathway that is involved in cell differentiation [44]. miR-21 is an inhibitor of T-cell apoptosis and involved in the skin inflammation component of psoriasis [45]. miR-31 has been shown to be regulated by transforming growth factor beta 1 (TGF-β1), a cytokine associated with psoriasis [45].

Besides miRNAs, accumulating evidence has recently shown that lncRNAs, one kind of non-protein coding transcripts longer than 200 nucleotides, play important roles in epigenetic regulations [46]. Tsoi et al. used computational approaches and identified 2942 previously-annotated and 1080 novel skin-specific lncRNAs [47]. Some novel lncRNAs are differentially expressed in psoriasis lesions versus uninvolved or normal skin. These lncRNAs are co-expressed with genes related to immune functions and enriched in the epidermal differentiation complex [47]. These results suggest that many lncRNAs might be involved in the pathogenesis of psoriasis.

Epigenetic alterations of DNA can affect gene expression. Global DNA methylation profiling showed that DNA methylation in psoriatic PBMCs is significantly increased compared to normal controls [41]. Some methylation-sensitive genes, including *LFA-1*, *SHP-1*, and P16^INK4α^, were aberrantly expressed in psoriasis patients [41]. Roberson et al. [48] investigated global CpG methylation in psoriasis and showed that methylation status at more than 1000 CpG methylation sites was different in psoriatic skin lesions compared with normal skin. There are inverse correlations between expression of nearby genes and methylation at these sites, including *KYNU*, *OAS2*, *S100A12*, and *SERPINB3*, whose strong transcriptional upregulation acts as a key indicator of psoriasis. Additionally, methylation levels could be restored to normal levels after anti-TNFα treatment [48]. These results suggest that prediction of therapy response using gene expression is feasible. Similar to methylation, histone modification is an epigenetic mechanism that affects gene expression through the modification of chromatin. In PBMCs from psoriasis vulgaris patients, hypoacetylation of histone H4 was observed and the degree of hypoacetylation was inversely correlated with PASI scores [49].

The current results suggest that epigenetic alterations including DNA methylation, histone modification, and expression of miRNAs/lncRNAs may play critical roles in psoriasis. However, their roles must be further confirmed by utilizing animal models and/or cell lines that carry loss-of or gain-of-function mutations. The relationship between epigenetic alterations and the pathogenesis in psoriasis is still unknown, although a variety of epigenetic alterations in psoriasis have been described.

## Proteomic biomarkers

Proteomics is the extensive and large-scale study of proteins in complex biological samples, particularly their structures and functions. Some biomarkers for psoriasis discovered by proteomic technology have been reported in recent years (Table 2). In 2004, the first study of psoriasis utilizing proteome analysis was performed to examine patterns of global protein expression from lesional and non-lesional skin of subjects with chronic plaque psoriasis and acute guttate psoriasis using 2-dimensional gel electrophoresis (2D-GE) with mass spectrometry (MS) [50]. Expression of 12 proteins was found to be upregulated more than 2 folds, including squamous cell carcinoma antigen (SCCA), cytokeratin14, cytokeratin17, and RhoGDI 1, or downregulated (*e.g.*, cytokeratin15 and calreticulin) in the psoriasis group compared with normal skin [50]. Using Multi-lectin affinity chromatography (M-LAC) followed by analysis with nanoscale liquid chromatography coupled to tandem MS (nanoLC-MS/MS), Plavina et al. [51,52] used two proteomic methods to analyze plasma samples from psoriasis patients and found that plasma concentrations of both cytoskeletal and Ca^2+^-binding proteins, as well as their peptides, were increased in psoriasis patients.

In order to tackle issues pertaining to protein complexity and highly-dynamic range prior to analysis, Williamson et al. [53] utilized keratome skin biopsy and *ex vivo* culture to enrich for “secretome” sub-proteome biomarkers reflective of the disease. Over 50 proteins frequently altered in high quantities in lesional *vs.* non-lesional psoriatic skin were identified with LC–MS/MS. These include multiple canonical psoriasis-related proteins such as S100A7 (psoriasin) and epidermal fatty acid binding protein (FABP5), as well as alteration in expression of over 30 novel proteins, such as profilin 1, galectin-related protein, and glutaredoxin-1. Another proteomic analysis with 2D-GE and LC–MS/MS found an up-regulation of glutathione S transferase pi 1(GSTP1), stratifin (SFN), and peroxiredoxin 2 (PRDX2) in psoriatic skin tissue [54]. Upregulated GSTP1 and PRDX2 in psoriasis might be explained by their important roles in preventing and protecting against DNA damage or cell death induced by reactive oxygen species ROS [54]. Piruzian et al. found 10 proteins with a 2-fold or greater increase in expression in lesional skin as compared with non-lesional skin, by combining 2D-GE and MS. Upregulation of these proteins (keratin 14, keratin 16, keratin17, SCCA, SCCA-2, enolase 1, superoxide dismutase, galectin-7, S100A9, and S100A7) were associated with notable overexpression of their respective coding genes [19].

Schonthaler et al. [55] performed unbiased proteomic analyses of human psoriatic epidermis and also found S100A8-S100A9 (calprotectin) to be the most upregulated proteins. Similarly, Lundberg et al. [56] used an unbiased proteomics screening approach to study changes in protein expression in the KC-Tie2 psoriasis mouse model. They further validated these changes in human psoriasis samples. In total 105 proteins exhibited fold-change ⩾2.0, including stefin A1, slc25a5, serpinb3b, and kallikrein related peptidase 6 (KLK6). In agreement with this study, increased gene expression of *slc25a5*, *cystatin A*, *KLK6*, and *serpinB1* was observed between healthy controls and involved lesional psoriatic skin and primary psoriasis keratinocytes [56].

After long-term use of MTX in psoriasis patients, hepatic fibrosis is a common adverse drug reaction. Urinary proteins were analyzed by matrix-assisted laser desorption/ionization time-of-flight MS (MALDI-TOF MS) and identified by electrospray ionization LTQ Orbitrap MS [57]. In the urine of psoriasis patients with a high cumulative MTX dose, some proteins that are known to be associated with hepatic fibrosis were identified, including haptoglobin, N-cadherin, serotransferrin, and inter-alpha-trypsin inhibitor heavy chain H4 [57]. These proteins may prove to be good candidate biomarkers for monitoring MTX-induced hepatic fibrosis in psoriasis patients [57].

Among all of these proteomic biomarkers, a few proteins like S100A8, S100A9, galectin 3 binding protein (G3BP), and profiling 1 have been validated using orthogonal methods or in large cohorts of patients and have also been confirmed by others [58]. However, the majority of potential proteomic markers have not been validated in animal models or human psoriasis. While there is a great deal of information about these molecules, less is known about the mechanisms that these molecules are driving. Further studies are warranted to tackle the molecular mechanisms of psoriasis and this will be crucial for identifying potential therapeutic targets.

## Metabolomic biomarkers

Metabolomics is an emerging approach in the field of systems biology [59]. However, thorough studies and data at the intersection of metabolomics and psoriasis are currently limited. Sitter et al. [60] examined and compared metabolic patterns between unaffected skin and psoriatic skin, as well as corticosteroid-treated psoriatic skin with 1D ^1^H nuclear magnetic resonance (NMR) spectroscopy. They found lower metabolite levels of glucose and myo-inositol, but higher levels of taurine and choline in tissue biopsies from psoriatic skin compared to unaffected skin. Tissue levels of glucose, myo-inositol, glycerol phosphorylcholine (GPC), and glycine were elevated in corticosteroid-treated psoriatic skin, whereas choline levels were decreased with good therapeutic effect.

Armstrong et al. [61] compared circulating metabolites in blood serum samples from patients with psoriasis or psoriatic arthritis, and healthy controls using a global metabolomics approach. Metabolite levels were measured by calculating the mean peak intensities from gas chromatography (GC) TOF-MS. Multivariate analyses of metabolomics revealed that psoriasis patients had higher levels of alpha ketoglutaric acid (AKG), and lower levels of asparagine and glutamine. Moreover, patients with both psoriasis and psoriatic arthritis had an increased level of lignoceric acid and a decreased level of AKG, compared to patients with psoriasis alone.

Recently, Kamleh et al. [62] performed a non-targeted high-resolution LC–MS metabolomics analysis to measure plasma metabolites from individuals with mild or severe psoriasis and healthy controls. They identified significant psoriasis-associated perturbations in three metabolic pathways: (1) proline and arginine, (2) glycine, threonine, and serine, and (3) aspartate, alanine, and glutamate. After treatment with the anti-TNFα drug Etanercept, the majority of psoriasis-associated alterations in circulating metabolites were reversed, shifting the metabolic phenotypes of severe psoriasis toward that of healthy controls. Circulating metabolite levels pre- and post-Etanercept treatment were correlated with PASI scores. These data suggest that levels of circulating amino acids are useful for monitoring both the severity of psoriasis disease and therapeutic responses to anti-TNFα treatment.

Although some potential biomarkers have been identified using various approaches spanning genomics, proteomics, and metabolomics, greater in-depth bioinformatics utilization will be pivotal in identifying the connections between these molecular signatures or pathways and in linking the data between “omics” technologies. In turn, this will grant the scientific/medical community a larger and more thorough picture of whether and how these molecules play key roles in the pathogenesis of psoriasis. Moreover, new insights into the molecular mechanisms of psoriasis will be gleamed from increasingly-integrated information and will likely result in significantly-improved clinical management of the disease.

## Technological challenges and opportunities

As the search for biomarkers continues, new technologies might facilitate the discovery of high-efficacy biomarkers in a comprehensive and unbiased manner. Traditional DNA sequencing technology used in laboratories has been hampered by their inherent limitations in throughput, speed, and scalability. In 2005, GWAS made its debut with the identification of a major susceptibility gene for a complex trait [63] and has become a transformative technology [63]. However, GWAS has its own limitations as well, including its restriction to common variants, incomplete genome coverage, and the inherent challenge of discerning the actual causal genetic variant [63]. An entirely new technology, next-generation sequencing (NGS) appeared, which can overcome these limitations. Much larger quantities of DNA fragments can be synthesized in parallel with NGS, allowing for the rapid sequencing of long DNA stretches and the measurement of variation across the entire genome [63].

Proteomic technologies have not experienced the same rapid improvements as genomics and a variety of challenges still exist. Traditional gel-based proteomics have been widely used in biomarker discovery but are limited by poor separation of acidic, basic, hydrophobic, and low abundance proteins. MS has advanced remarkably in the past decade. It can be coupled with increasingly-powerful technologies such as ion mobility separations, or microchip proteomic measurements with nanoscale reversed phase LC and capillary electrophoresis (CE) [64]. However, there are still many challenges when using MS for identifying, describing, and quantifying proteins, including sensitivity, specificity, reproducibility, and cost. Methods that utilize antibodies are known to be more sensitive as compared to 2D gels or MS [65]. Due to the high affinities of antibodies to their targets, these methods can even detect analytes down to the sub-nM range (usually nM to pM) [66]. A variety of antibody-coated protein microarrays have been utilized in the study of autoimmune diseases such as lupus [67–69]. However, antibody-based protein arrays are limited by the availability of high-quality antibodies.

Recently, multiple reaction monitoring (MRM) has become an emerging MS technique based on the selection of a peptide ion and one or more characteristic fragment ions [70]. The MRM method has been shown to be specific, accurate, and reproducible between laboratories. MRM-based quantitation can also be multiplexed to analyze and quantify hundreds of proteins per run, increasing the throughput of this type of assay and making it fast enough for clinical applications. MRM is particularly important for validation studies, where specific antibodies are unavailable for antibody-based assays, such as ELISA, Western blot, and IHC. Another emerging proteomic technology is the SOMAscan proteomics platform, which can efficiently, accurately, and rapidly identify and quantify over 1000 proteins across a wide range of concentrations (while targeted analysis is more suitable for examining specific metabolic pathways) in small sample volumes [71]. Compared to other proteomic technologies, SOMAscan may offer unprecedented power for discovering biomarkers due to its breadth and depth of coverage.

Metabolomics, a field emerging more recently relative to proteomics or genomics, examines the interactions, structures, and concentrations of small molecules/metabolites in biological systems [72], that is, the downstream products of genomics, transcriptomics, and proteomics [73]. Notably, metabolites can now be profiled at the single cell level [74]. Given minor stimuli could result in profound physiological changes, it is crucial to ensure consistency, minimalized interindividual discrepancies, and enhanced information recovery when designing metabolomic studies [73]. The most challenging task in metabolomics is to confirm the identity of a biomarker. Studies can be classified as non-targeted and targeted metabolomics depending on the experimental methods [75]. Non-targeted metabolome analyses are often preferred for their suitability in non-biased metabolite identification and biomarker exploration, while targeted analysis is more suitable for examining specific metabolic pathways. Nevertheless, significant issues arise due to the fact that metabolites vary widely in electrical charge, molecular weight, and concentration.

The main analytical platforms for both *in vitro* and *in vivo* studies are NMR and MS, respectively. NMR is useful for analyzing molecules such as sugars, amines, and volatile liquids [73]. Technical advances, such as cryoprobes and higher magnetic field strengths, have enhanced spectral dispersion and increased sensitivity to the nM range, while the identification of more specific metabolite species has been improved via statistical recovery techniques, such as kinetic and j-resolved total correlation spectroscopy [76], which boost signal dispersion and overall information recovery [73]. Furthermore, a large range of protein and lipoprotein signals can be mitigated through various pulse sequences [73]. Nonetheless, NMR comes with the higher start-up costs, requires larger sample volumes and is overall less sensitive than MS techniques. MS separation techniques most commonly include GC and more recently, ultra-high performance LC (UHPLC), as well as CE for complementary information. Although UHPLC–MS and GC–MS both have advantages over other metabolomic technologies, such as higher resolutions and lower costs, many challenges currently remain [73]. For example, UHPLC–MS is destructive to samples, while GC–MS samples require extensive preparation and samples must be volatile. Furthermore, the results of all MS techniques in metabolomic studies using the same or similar samples are difficult to compare and correlate between independent research groups due to the measurement dependence on analytical platforms, methods, and protocols [77].

Newer technological advances in metabolomics show promising features in specificity and integration. Nanostructure-initiator MS (NIMS) analyzes metabolites using a desorption and ionization approach, creating little fragmentation and requiring no matrix or sample preparation [73]. In addition, metabolic flux can be monitored with stable isotope tracers to elucidate further metabolic networks [73]. Furthermore, computational and collaborative technologies are rapidly improving [73] to help cope with complexities in metabolomics research, such as individual variations and metabolite influences from the gut microbiome. For instance, the first entirely open-source online platform for computational metabolomics, Workflow4Metabolomics, was recently developed [78].

Technological advances are also leading to increased integration across the full spectrum of “omics” research. One notable example of a new combinatorial method is *in vitro* virus-high-throughput sequencing (IVV-HiTSeq) [75]. The IVV method utilizes puromycin to covalently bind mRNA to its encoded protein, and the IVV is synthesized from cDNA with a cell-free translation system [79]. IVV-HiTSeq combines IVV with NGS, and can generate high quantities of accurate protein (domain) interaction data under cell-free conditions [75]. Yet these data are not limited to protein–protein interactions, and may also include protein–RNA/DNA interactions, as well as protein–chemical compound interactions [75]. The massive amount of data generated from the genome, transcriptome, proteome, and metabolome is designated as the integrome, and likewise, its complex combinatorial properties are studied through the lens of interactome research. Therefore, as technology continues its rapid progression, techniques will become increasingly integrated and overlapping. Consequently, different platforms for biomarker discovery will become interactive and complementary eventually granting significant insights into human physiology and pathophysiology altogether.

## Conclusions

Here we have summarized the latest findings for potential biomarkers in psoriasis and discussed various “omics” technologies that have been used in biomarker discovery for psoriasis. Some of these markers might have clinical diagnostic and/or therapeutic potentials (Figure 1). On the other hand, it is also important to note that most of these markers are universal for inflammation instead of being specific to psoriasis. In addition, a key element for translating laboratory biomarkers into clinical applications, aside from the robustness of the scientific rationale involved, is the validation process. Valuable biomarkers should be validated using orthogonal techniques, such as ELISA, Western blot, qRT-PCR, and IHC with a larger cohort of subjects. This step creates a potential roadblock in the search for ideal psoriasis biomarkers that may be fully utilized in a clinical manner, and likewise, a great deal of work still lies ahead. Despite this, the accelerating emergence, availability, application, and convergence of high-precision, high-throughput “omics” technologies, and sophisticated bioinformatics will continue to open new avenues to the discovery of novel and specific psoriasis biomarkers with significant diagnostic and/or therapeutic values.

## Competing interests

The authors have declared no competing interests.

## Figures and Tables

**Table 1:** Major non-MHC psoriasis gene variants identified by GWAS

**Gene/locus**	**Chromosomal location**	**Proposed function**	**Ref.**
*IL-23A*	12q	IL-23/Th17 axis	[28]
*IL23R*	1p	IL-23/Th17 axis	[29]
*IL12B*	5q	Th1 cell differentiation	[29]
*ZNF313*	20q	Ubiquitin pathway	[30]
*TNIP1*	5q	NF-κ B pathway	[31]
*TNFAIP3*	6q	NF-κ B pathway	[31]
*RUNX3*	1p	Th1 cell differentiation	[33]
*STAT3*	17q	Th17 cell differentiation	[33]
*TAGAP*	6q	T cell activation	[33]
*ZC3H12C*	11q	Macrophage activation	[33]
*CARD14*	17q	NF-κ B pathway	[33]
*CARM1*	19p	NF-κ B pathway	[33]
*DDX58*	9p	IFN production	[33]
*LCE3A/3C/3D*	1q	Skin barrier function	[34]

*Note:* ZNF, zinc-finger protein; TNIP1, TNFAIP3 interacting Protein 1; TNFAIP3, TNF-α-induced protein 3; RUNX3, Runt-related transcription factor 3; TAGAP, T-cell activation Rho GTPase activating protein; ZC3H12C, Zinc finger CCCH-type containing 12C; CARD14, caspase recruitment domain family, member 14; CARM1, coactivator-associated arginine methyltransferase 1; DDX58, DEAD (Asp-Glu-Ala-Asp) box polypeptide 58; LCE, late cornified envelope.

**Table 2:** Proteomic biomarkers in psoriasis

**Samples**	**Methods**	**Proteins with altered expression**		**Ref.**
**Upregulated**	**Downregulated**
Skin tissue (stable chronic plaque psoriasis)	2D-GE, LC–MS/MS or MALDI-TOF	SCCA-2, maspin, cytokeratin14, cytokeratin17, GST-π, HSP27, 14-3-3*σ*	Cytokeratin10, cytokeratin15, calreticulin	[50]
Plasma sample (moderate to severe psoriasis)	M-LAC, nanoLC–MS/MS	G3BP		[51]
Plasma sample (moderate to severe psoriasis)	M-LAC, nanoLC–MS/MS	Thymosin β4, talin 1, actin γ, filamin, profilin, cytoskeletal, calgranulins A and B		[52]
Keratome biopsies (*ex vivo*) (plaque psoriasis)	Dimethyl labeling LC–MS/MS	Profilin 1, gasdermin A, PA2G4, CBR1/CBR3		[53]
Skin tissue (psoriasis)	2D-GE, LC–MC/MS	GSTP1, SFN, PRDX2		[54]
Skin tissue (plaque psoriasis)	2D-GE, MALDI-TOF MS	Galectin-7, S100A9/S100A7, keratin 14/16/17		[19]
Skin tissue psoriasis patients (PASIs > 15)	Unbiased iTRAQ	S100A8, S100A9		[55]
Skin tissue (KC-Tie2 psoriasis mouse model)	Gel-based label-free expression LC–MS/MS	Stefin A1, slc25a5, serpinb3b, KLK6		[56]

*Note:* “Upregulated” and “Downregulated” indicate the increased and decreased levels of the protein marker in psoriatic samples compared to normal control, respectively. PASI, psoriasis area and severity index; KC, keratinocytes; 2D-GE, 2-dimensional gel electrophoresis; LC, liquid chromatography; MS, mass spectrometry; MALDI-TOF, matrix-assisted laser desorption/ionization time of flight; M-LAC, multi-lectin affinity chromatography; iTRAQ, isobaric tags for relative and absolute quantitation; SCCA-2, squamous cell carcinoma antigen-2; GST-π, glutathione S transferase P; G3BP, galectin-3 binding protein; PA2G4, proliferation-associated protein 2G4; CBR, carbonyl reductase; GSTP1, glutathione S transferase pi 1; SFN, stratifin; PRDX2, peroxiredoxin 2; KLK6, kallikrein related peptidase 6.
